# The Coupling of Ferroelectric Polarization and Oxygen Vacancy Migration Enables Electrically Controlled Thermal Memories

**DOI:** 10.1002/adma.202519670

**Published:** 2026-03-30

**Authors:** Dídac Barneo, Miquel Royo, Rafael Ramos, Jesús Carrete, Hugo Romero‐Bernad, Ricardo Jiménez, Víctor Leborán, César Magén, Noa Varela‐Domínguez, Miguel Algueró, Riccardo Rurali, José A. Pardo, Francisco Rivadulla, Eric Langenberg

**Affiliations:** ^1^ Departament de Física de la Matèria Condensada Universitat de Barcelona Barcelona Spain; ^2^ Institut de Nanociència i Nanotecnologia (IN^2^UB) Universitat de Barcelona Barcelona Spain; ^3^ Institut de Ciència de Materials de Barcelona ICMAB‐CSIC Campus UAB Bellaterra Spain; ^4^ Centro Singular en Química Biolóxica e Materiais Moleculares (CiQUS) Universidade de Santiago de Compostela Santiago de Compostela Spain; ^5^ Departamento de Química‐Física Universidade de Santiago de Compostela Santiago de Compostela Spain; ^6^ Instituto de Nanociencia y Materiales de Aragón (INMA) CSIC‐Universidad de Zaragoza Zaragoza Spain; ^7^ Departamento de Física de la Materia Condensada Universidad de Zaragoza Zaragoza Spain; ^8^ Instituto de Ciencia de Materiales de Madrid (CSIC) Madrid Spain; ^9^ Departamento de Ciencia y Tecnología de Materiales y Fluidos Universidad de Zaragoza Zaragoza Spain; ^10^ Laboratorio de Microscopías Avanzadas, Campus Río Ebro Universidad de Zaragoza Zaragoza Spain

**Keywords:** ferroelectric hafnia‐based epitaxial oxides, oxygen vacancy migration, thermal conductivity, thermal memory

## Abstract

Here we investigate epitaxial Hf_0.5_Zr_0.5_O_2_ ferroelectric thin films as potential candidates to be used as non‐volatile electric‐field‐modulated thermal memories. The electric‐field dependence of the thermal conductivity of metal/Hf_0.5_Zr_0.5_O_2_/Y_2_O_3_:ZrO_2_ devices is found to be hysteretic—resembling a polarization vs. electric field hysteresis loop—, reaching a maximum (minimum) at large applied positive (negative) electric fields from the top metallic electrode. This dynamic thermal response is compatible with the effects of the coupling between the ferroelectric polarization and oxygen ion migration in the Hf_0.5_Zr_0.5_O_2_ layer, in which the oxygen vacancies are the main phonon scattering centers and the polarization acts as an electrically active ion migration barrier that creates the hysteresis. This new mechanism enables two non‐volatile states: high (ON) and low (OFF) thermal conductivity states when the electric field is removed, with an ON/OFF ratio of 1.6, which can be switched with applied voltages lower than ‐5 and +5 V, respectively. Both the ON and OFF states exhibit high stability over time, though the switching speed is limited by ion mobility in the Y_2_O_3_:ZrO_2_ electrode.

## Graphical abstract

Ferroelectric Hf_0.5_Zr_0.5_O_2_ epitaxial thin films exhibit a non‐volatile, electrically controlled thermal conductivity enabled by the coupling between oxygen vacancy migration, acting as phonon scatterers, and ferroelectric polarization, acting as ion migration valve. The resulting hysteretic thermal response allows reversible access to distinct thermal states, establishing hafnia‐based ferroelectrics as a promising platform for solid‐state thermal memories and advanced thermal management technologies.

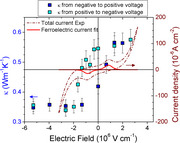

## 1 Introduction

The identification of materials whose thermal conductivity (*κ*) can be reversibly tuned using an external stimulus—such as electric [1, 2, 3, 4, 5, 6, 7, 8, 9], magnetic [10, 11, 12, 13], optical [14], thermal [3], strain [15], or electrochemical fields [16, 17, 18, 19, 20]—is a key step toward the realization of thermal memories. This is, a solid‐state device capable of reversibly switching between two distinct thermal states—high (*κ*_ON_ state) and low (*κ*_OFF_ state) thermal conductance—in a non‐volatile manner, such that the selected thermal state remains stable after removal of the external writing stimulus. By enabling the controlled opening and closing of heat transport pathways, thermal memories provide a means to actively route thermal energy on demand. This capability is relevant for applications such as thermal energy storage and management, where heat flow must be selectively directed or blocked, as well as for advanced thermoelectric systems in which heat can be dynamically steered toward thermoelectric modules to enhance energy harvesting efficiency [21, 22, 23, 24, 25, 26]. Thermal memories may also play a crucial role as thermal control elements in solid‐state refrigeration technologies based on caloric materials, where efficient heat management is essential to achieve high power density and energy efficiency [26, 27]. More broadly, thermal memories can function as thermal regulators in devices that require strict temperature stability, such as microprocessors or batteries, where excess heat generated by Joule dissipation can severely degrade performance, endurance, and energy efficiency. Beyond thermal management, continued progress in thermal memories may enable technologies for storing and processing information using heat currents rather than electrical currents [21, 26, 28, 29]. Such thermal‐based information processing platforms offer the potential for improved energy efficiency through heat reuse and recovery, particularly in systems where heat is otherwise an unavoidable byproduct [29, 30]. Finally, since phonons are the primary heat carriers in electrically insulating solids, thermal memories naturally contribute to the emerging field of phononics, which aims to develop logic, data storage, and computing architectures based on the controlled manipulation of phonon transport [31, 32, 33, 34, 35, 36, 37, 38, 39, 40].

Among all the previously mentioned trigger mechanisms to alternate between the *κ*_ON_ and *κ*_OFF_ states, the electric field is the easiest one to integrate in a solid‐state thermal memory, requiring basically just two electrodes and an applied voltage. In addition, an electric field can be applied locally, allowing a high density of “thermal bits.” Moreover, low voltage values can generate a sufficiently large electric field, especially in thin‐film solid‐state architectures, reducing the energy consumption [41]. In this regard, ferroelectric materials have played a pivotal role in electrically tuning *κ* thus far, relying on several different approaches: i) by electrically modifying the density of domain walls, which behave as active phonon scattering centers in PbZr_1‐x_Ti_x_O_3_ or BaTiO_3_ polycrystalline samples, and in PMN‐PT (Pb(Mg_1/3_Nb_2/3_)O_3_‐PbTiO_3_) single crystals [1, 2, 4, 9]; ii) by electrically inducing a phase transition from antiferroelectric to ferroelectric in PbZrO_3_ epitaxial films, where each phase has a different κ [3]; iii) by electrically switching between two types of ferroelectric‐ferroelastic domains, where a‐ and c‐domains display different values of *κ*, in single‐crystal BaTiO_3_ [8]; and iv) by electrically changing the phonon dispersion and, thus κ through the phonon velocities in single‐crystal PbZr_1‐x_Ti_x_O_3_ [7]. The main advantage of using ferroelectrics, particularly in electrically modulating the domain wall density, is the rapid *κ*_ON_/*κ*_OFF_ switching, which tends to be in the ns timescale [28]. However the *κ*_ON_/*κ*_OFF_ ratio is in general quite low, typically ranging from 1.1 to 1.2 [1, 2, 3, 4, 7, 9], except for the phase transition from antiferroelectric to ferroelectric in PbZrO_3_ epitaxial films, in which a 2.2 ratio has been reported [42]. In this latter case, though, the *κ*_ON_ state—corresponding to the ferroelectric phase—is volatile: it only exists while the electric field is applied [42]. Once the field is removed, the antiferroelectric phase—corresponding to the *κ*_OFF_ state—is immediately recovered [42], inhibiting it from being used as a non‐volatile memory bit. Actually, this volatile nature of the *κ*_ON_ states is commonly encountered in the “ferroelectric approaches” [28].

One of the strategies that have achieved much larger *κ*_ON_/*κ*_OFF_ ratios (up to 5.4) is the perovskite (*x* = 3)—brownmillerite (*x* = 2.5) topotactic phase transformation via oxygen ion migration in epitaxial SrCoO_x_, La_0.5_Sr_0.5_CoO_x_, and (Ca,Sr)FeO_x_ films [16, 17, 18, 20]. The brownmillerite structure displays much lower *κ* than the perovskite structure, which might be due to the large amount of oxygen vacancies acting as point defect phonon scattering centers [17]. In fact, both cation and anion defects are extremely efficient in reducing *κ* in oxide materials [43, 44, 45, 46]. Moreover, this oxygen migration can be triggered electrically at 280°C using yttria‐stabilized zirconia (YSZ, Y_2_O_3_:ZrO_2_) substrates as solid electrolytes [17, 20], without the need of ionic liquids [16, 18, 19] that hamper their integration in solid‐state structures. Furthermore, the endurance of the perovskite‐brownmillerite phase transition cyclability is substantially improved by the solid electrolyte compared to the use of ionic liquids or oxidizing gas atmospheres, which tends to damage the structure after a couple of cycles [20]. However, the perovskite‐brownmillerite phase transition takes several minutes [16, 17, 18, 20, 28], which entails a much slower *κ*_ON_/*κ*_OFF_ switching time than previous approach using electrically mobile domain walls in ferroelectrics. Recently, a voltage‐biased atomic force microscopy tip was used to drag oxygen vacancies in SrFeO_3‐x_, La_0.6_Sr_0.4_CoO_3‐x_ and La_0.7_Sr_0.3_MnO_3_ epitaxial films on SrTiO_3_ substrates at room temperature, locally writing non‐volatile low thermal conductivity states [47]. Still, to switch back to the as‐grown thermal conductivity state (i.e. to erase the low thermal conductivity states) temperature, instead of voltage, was required, inhibiting a full electric field control of the *κ*_ON_/*κ*_OFF_ switching [47].

Related behavior has been reported in oxide‐based dielectric memristive systems, where electrically driven redistribution of charged defects is known to strongly affect both electrical and thermal transport. In particular, reversible modulation of interfacial thermal resistance has been demonstrated in metal/oxide memristors, where accumulation and depletion of oxygen vacancies at the metal/oxide interface—induced by the polarity of the applied electric field—led to changes of up to ≈20% in thermal resistance at Pt/SrTiO_3_ and Cr/SrTiO_3_ interfaces [48]. More recently, large and reversible variations in the thermal conductivity of CeO_2_ films have been reported as a consequence of repeated oxygen exchange with YSZ by applying constant electrical current—similar to memristors, where current is used to control charge defects in the dielectric layer [49].

Here, we propose the use of ferroelectric Hf_1‐x_Zr_x_O_2_ as the electrically active thermal barrier, to take advantage of the close relationship between the stabilization of the metastable polar phase and the local concentration of oxygen vacancies [50, 51, 52]. On the one hand, ferroelectric Hf_1‐x_Zr_x_O_2_ intrinsically contains anion defects [16, 17, 18]. On the other hand, the oxygen vacancy migration through epitaxial ferroelectric Hf_0.5_Zr_0.5_O_2_ (HZO) films grown on La_0.67_Sr_0.33_MnO_3_/Nb:SrTiO_3_ and using non‐reactive metals like Au as top electrode has been proven to be quite fast (of the order of seconds) [53].

## 2 Results and Discussion

**FIGURE 1:**
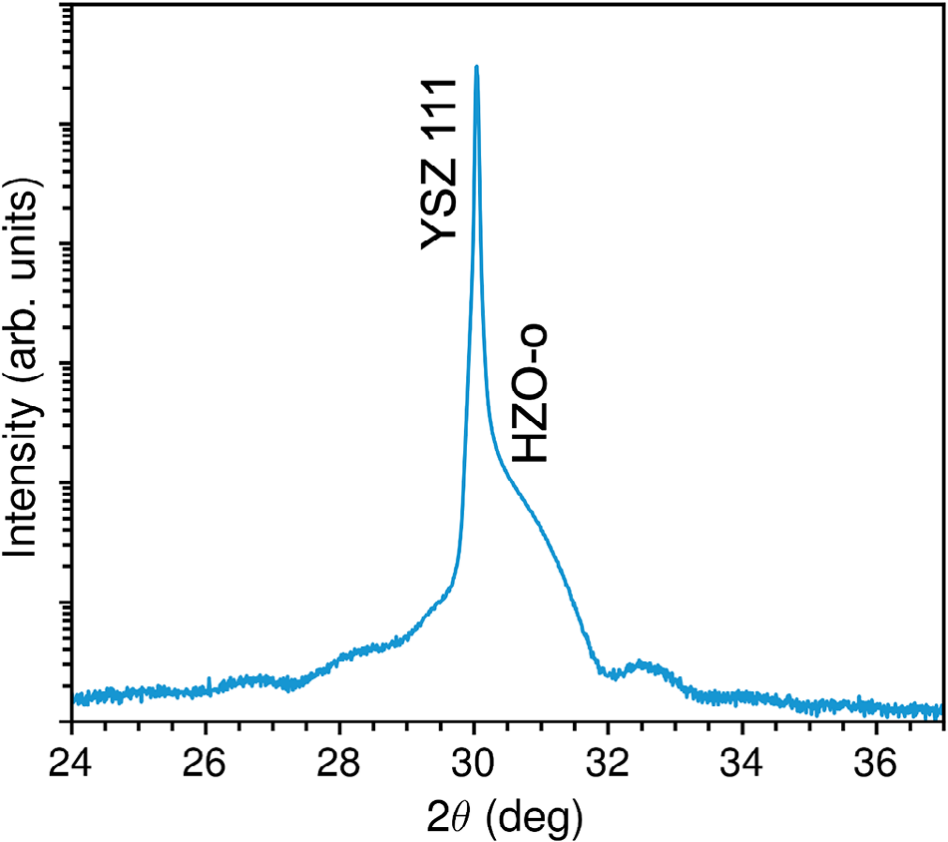
X‐ray diffraction pattern of Hf_0.5_Zr_0.5_O_2_ films deposited onto 111‐oriented YSZ substrates.

**FIGURE 2:**
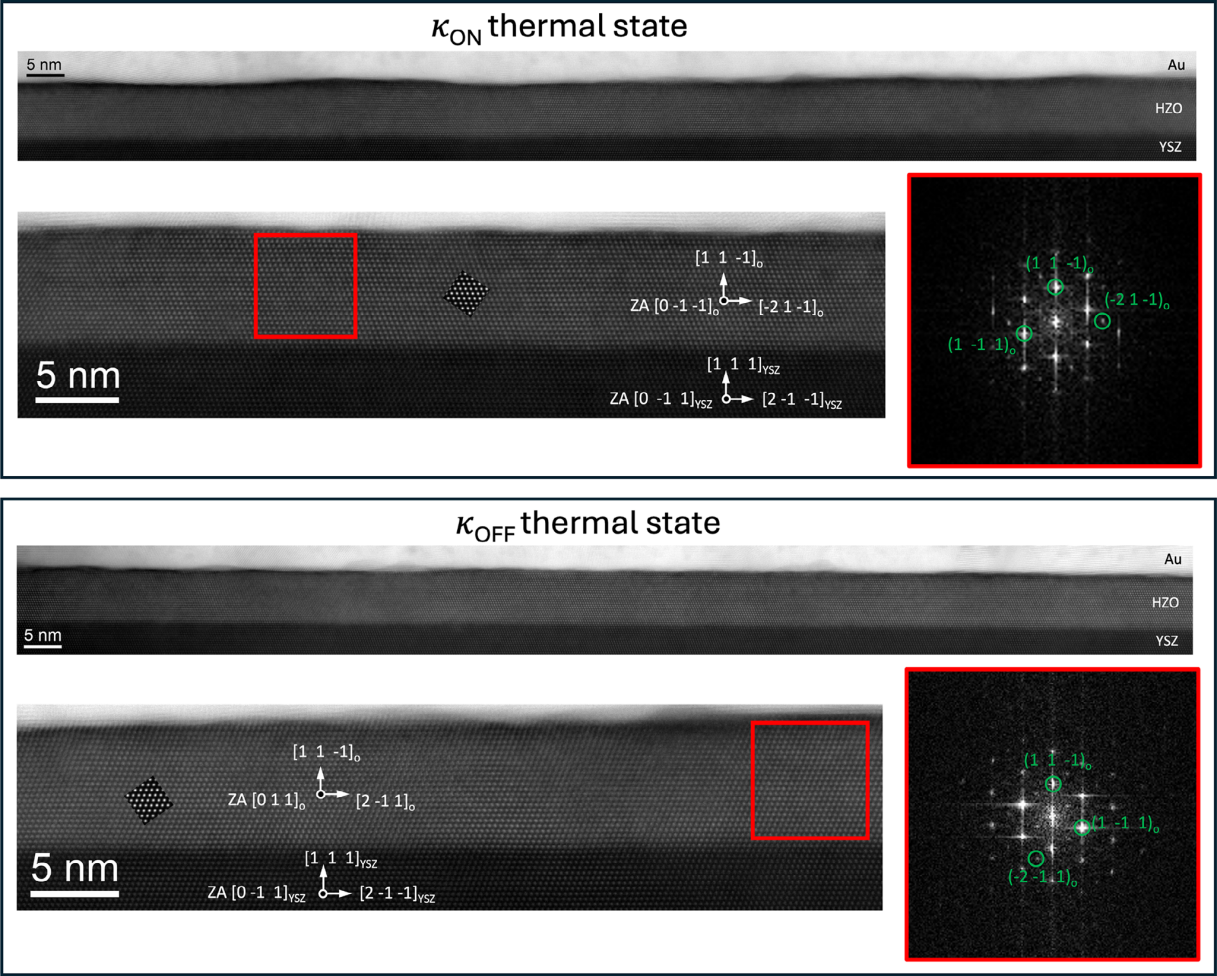
Atomic resolution cross‐sectional HAADF‐STEM images of the Au/HZO/YSZ(111) heterostructure. Top panel: device polarized with a positive bias of +4 V in the *κ*_ON_ state. Bottom panel: device polarized with a negative bias of ‐4 V in the *κ*_OFF_ state. Fast Fourier transform (FFT) of the regions marked with red squares are presented, and have been indexed according to the orthorhombic structure *Pca*2_1_ in both *κ*_OFF_ and *κ*_ON_, states, confirming that the ferroelectric phase remains unaltered by the oxygen vacancies migration. The epitaxy relations of YSZ and HZO deduced from the FFTs are overlayed, and STEM image simulations along the corresponding zone axis are included as insets in the HZO layer.

To assess this potential functionality of ferroelectric HZO as a thermal memory, 7 nm thick epitaxial HZO films were grown on isomorphic 111‐oriented YSZ substrates by pulsed laser deposition (PLD); the growth conditions (see Experimental Section) are reported elsewhere [54]. Note that the use of single‐crystal substrates of the same fluorite structure as the epitaxial film that is to be deposited ensures coherent growth of the films and the minimization of grain boundaries or structure defects, and avoids the coexistence of different polymorphs that would act as electrically inactive phonon scattering centers, reducing the effectiveness of the *κ*_ON_/*κ*_OFF_ switching. In addition, YSZ intrinsically contains a large concentration of oxygen vacancies and behaves as an oxide‐ion conductor [17, 20, 54], hence acting as a sink and source for the oxygen‐vacancy migration from/to the HZO film. The epitaxial strain exerted by the (111)‐oriented YSZ substrates stabilizes the polar structure in the HZO films, with no traces of the non‐polar monoclinic structure, as shown in the X‐ray diffractograms around the 111 substrate reflection (Figure 1 and Figure S1). Moreover, the Laue fringes around the 111 film reflection (Figure 1) indicate a high crystal quality of the films, as discussed in a previous work [54], which is further corroborated by atomic‐scale characterization of cross‐section lamellae using scanning transmission electron microscopy (STEM) shown in Figure 2.

**FIGURE 3:**
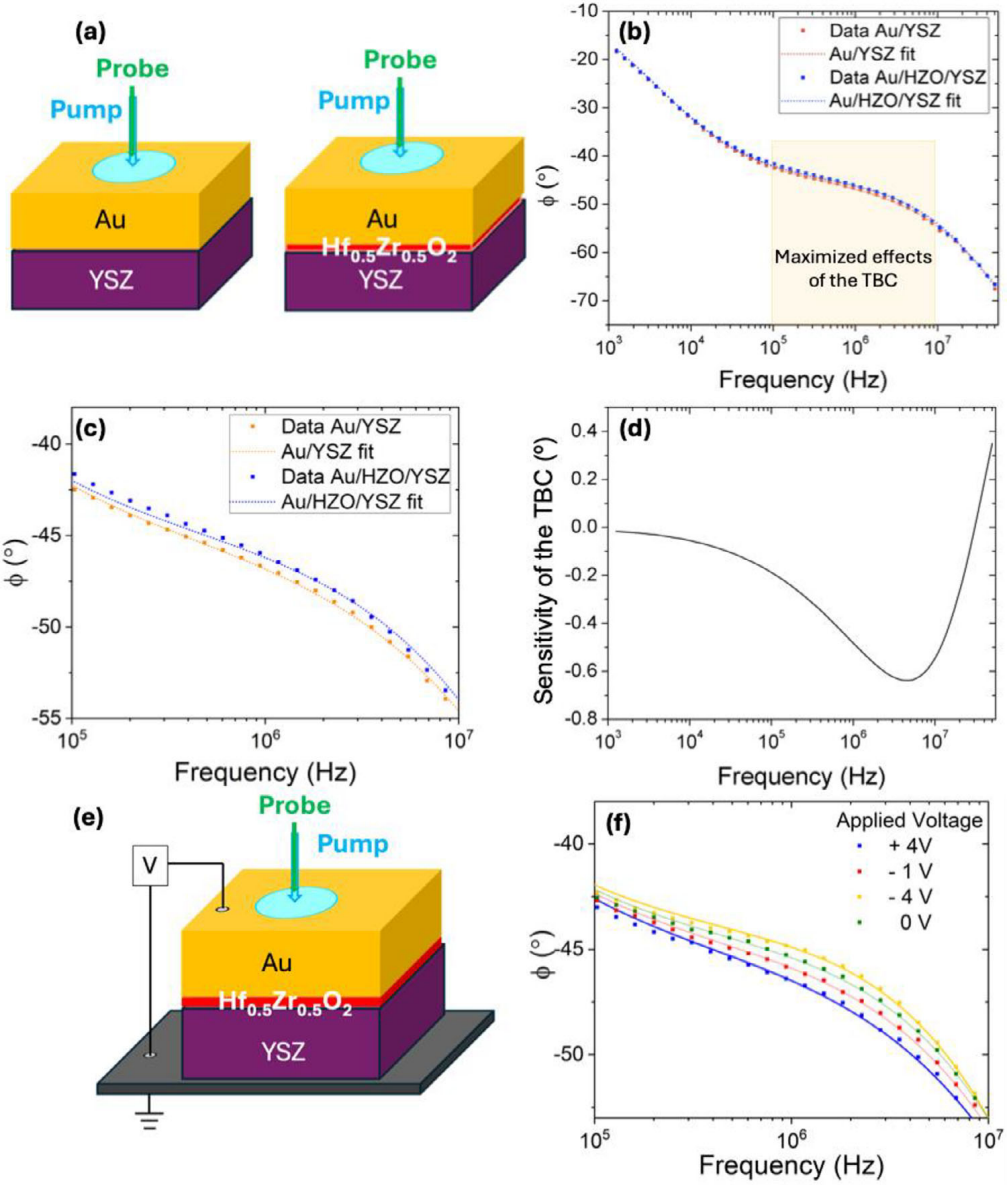
FDTR measurements. (a) Sketch of the FDTR measurements of the Au/Hf_0.5_Zr_0.5_O_2_/YSZ and Au/YSZ samples. Hf_0.5_Zr_0.5_O_2_ is modelled as an interface thermal boundary conductance (TBC) as the Au/YSZ interface. (b) Frequency‐dependent phase (*ϕ*) data of the FDTR measurements at room temperature. The frequency range where the effects of the presence or absence of the Hf_0.5_Zr_0.5_O_2_ film are observed is shadowed in yellow. (c) Zoom‐in at the frequency range where the sensitivity of the interfacial TBC parameter is the largest (d) Sensitivity analysis of TBC. The graph corresponds to the phase shift that occurs when changing the TBC parameter by 10%. (e) Sketch of the FDTR measurements performed while applying an electric field on the Au/Hf_0.5_Zr_0.5_O_2_/YSZ samples. (f) Frequency‐dependent phase (*ϕ*) data of the FDTR measurements at different electric fields at 200°C on the Au/Hf_0.5_Zr_0.5_O_2_/YSZ samples.

Non‐reactive Au, in the form of 60 nm‐thick pads, was ex‐situ deposited on top of the HZO/YSZ samples by sputtering, acting as top electrodes and transducers for the frequency‐domain thermoreflectance (FDTR) to measure the thermal properties under different applied electric fields (see Methods and Supporting Information) [55, 56, 57]. The thermal properties were obtained by fitting the frequency( *f* )‐dependent phase data, *ϕ*( *f* ), of the FDTR experiments to an analytical solution of the heat diffusion equation in a multilayer model [56]. Due to the low thickness value of the HZO film (7 nm), the whole HZO layer is modelled as a thermal boundary conductance (TBC, Figure 3a) between the Au layer and the YSZ substrate (see further details in the Supporting Information). The comparison between the raw phase‐shift data recorded at room temperature for Au/HZO/YSZ and Au/YSZ shows that the insertion of the HZO film results in an increase of the phase at intermediate frequencies (Figure 3b,c), corresponding to the frequency range where the sensitivity of the TBC is the largest (Figure 3d). This is consistent with a decrease of the TBC of the device due to the presence of the HZO film. By determining the TBC between the Au film and YSZ in the Au/HZO/YSZ stack using the heat diffusion equation in a multilayer model [56], the *κ* of the HZO film is computed by multiplying the TBC by the thickness (see further details in the Supporting Information). A similar approach was already considered in earlier work [58]. Next, the FDTR measurements were performed at 200°C on the Au/HZO/YSZ sample while applying different electric fields from the top electrode (Au) and using YSZ as grounded bottom electrode (Figure 3e). Some representative experimental *ϕ*(*f*) data (solid squares) at different applied electric fields and the corresponding fitting (solid lines) to the solutions of the heat diffusion equation in a multilayer model are shown in Figure 3f and Figure S3 [55, 56]. As observed, the *ϕ*(*f*) data in the previously mentioned frequency range (between ≈10^5^ Hz and ≈10^7^ Hz) in which the effects of the TBC are maximized significantly changes with the applied electric field, which indicates that the TBC of the ferroelectric HZO layer is strongly electric‐field‐dependent. Note that electric field dependent FDTR measurements were also carried out in the Au/YSZ samples—i.e., without the HZO layer—and in Au/HZO_m_/YSZ samples—where HZO_m_ stands for non‐polar monoclinic HZO layer (see further details in Supporting Information and Figure S1)—in which no noticeable change in the *ϕ*(*f*) data is observed (see Figures S3 and S4).

**FIGURE 4:**
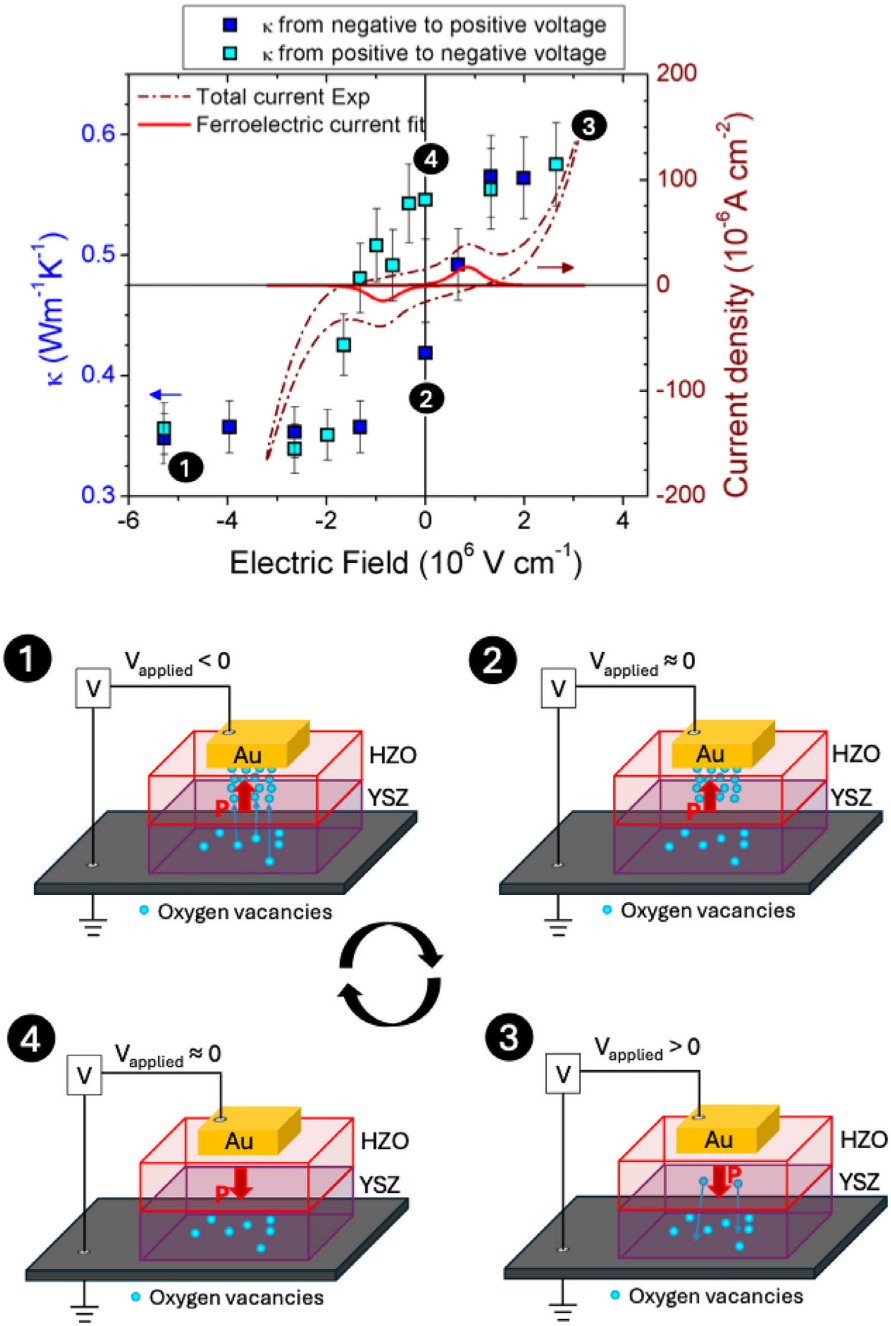
Top panel. Left axis: Electric‐field dependence of the thermal conductivity of Hf_0.5_Zr_0.5_O_2_ films at 200°C. The numbers 1, 2, 3, and 4 refer to the sketch in bottom panels. Right axis: current–electric field measurements at 185°C and at 0.01 Hz (dashed red line). After subtracting both the leakage and capacitive contributions, we isolate the ferroelectric switching current (solid red line). Bottom panels. Sketch showing the coupling mechanism between oxygen‐vacancy migration and polarization in Hf_0.5_Zr_0.5_O_2_ films and its effects on the *κ*(*E*) hysteresis loop.

The resulting *κ* values of the HZO layer are shown in Figure 4 (top panel, left axis) and they vary from a minimum of ≈ 0.35 W m^−1^ K^−1^ to a maximum ≈0.55 W m^−1^ K^−1^ depending on the applied electric field. These values are slightly below the reported *κ* in 20 nm thick Hf_1‐x_Zr_x_O_2_ films (*κ* ≈ 0.7–1.2 W m^−1^ K^−1^) [58], due to the reduced thickness of our films. Indeed, for a hypothetic bulk single‐crystal ferroelectric HZO sample, a much larger *κ* has been predicted (≈ 4 W m^−1^ K^−1^) [59]. The dependence of *κ* with the applied electric field was measured by gradually varying the field from negative to positive and back from positive to negative. Since no change was noticed when increasing the field above 2·10^6^ V cm^−1^, we started to go back to negative fields from there (hence the asymmetry of the electric‐field measuring range). Strikingly, the electric field dependence of *κ*, *κ*(*Ε*), is clearly hysteretic in our HZO films (Figure 4, top panel, left axis). To the best of our knowledge, *κ*(*E*) hysteresis loops have never been reported in a ferroelectric material. This result enables the definition of two non‐volatile thermal conductivity states (*κ*_ON_ and *κ*_OFF_ for the high and low thermal conductivity states, respectively).

To compare this thermal response with the regular ferroelectric polarization‐electric‐field, *P*(*E*), hysteresis loop, current–electric field measurements on the Au/HZO/YSZ samples were performed (Figure 4, top panel, right axis, dashed dark red line)—see Experimental Section. The resulting current loop exhibits two well‐defined ferroelectric switching peaks at approximately ±0.85 × 10^6^ V cm^−^^1^, confirming the ferroelectric nature of HZO and identifying the coercive electric fields. After subtracting both the leakage and capacitive contributions, we isolate the ferroelectric switching current (Figure 4, right axis, solid red line), from which the *P*(*E*) hysteresis loop is reconstructed (Figure S6). Notably, the coercive fields extracted from the *κ*(*E*) hysteresis loop coincide reasonably well with the ferroelectric switching peaks observed in the current–electric field measurements, thereby inferring a relationship between *κ*(*E*) and *P*(*E*) responses as discussed next.

The *κ*(*E*) hysteresis loop shown in Figure 4 is not consistent with a scenario where domain walls are the main phonon scattering mechanism, as reported in conventional ferroelectrics [1, 2, 4, 9]. If that were the case, the *κ*(*E*) response should be quite different: the minimum *κ* should be around the coercive fields, when the density of domain walls is large; conversely, *κ* should be maximum at both large positive fields and large negative fields, when the ferroelectric film is single domain and thus domain walls are absent. Here, instead, *κ* is minimum at large negative electric field and maximum at large positive electric field. Therefore, although phonon scattering by domain walls in HZO cannot be completely ruled out, they cannot be the main contributors to the observed *κ*(*E*) response. This contrasts with the notable effect that domain walls have on the thermal conductivity of perovskite ferroelectrics [15]. In these materials, domain walls typically extend over several unit cells, whereas in hafnia‐based ferroelectrics they are often atomically sharp and confined to sub‐nanometer widths [60]. This extremely small spatial extension is expected to substantially reduce their effectiveness as phonon scattering centers compared to the much broader domain walls found in perovskite oxides.

Alternatively, given the different polymorphs in which hafnia‐based oxides can be stabilized [50, 51, 52]—particularly the role of oxygen vacancies in tipping the balance between the monoclinic and orthorhombic phases—it is possible that the electric field triggers a phase transition in our HZO films via oxygen vacancy migration, thereby changing their thermal conductivity. However, STEM analysis of cross‐sectional lamellae from the Au/HZO/YSZ samples in both the *κ*_ON_ and *κ*_OFF_ states (Figure 2) reveals that the HZO phase remains orthorhombic, regardless of the thermal state, with no monoclinic regions. Note that to perform the STEM measurements at room temperature, the samples were cooled down while applying the negative electric field for the *κ*_OFF_ states evaluation and positive for the *κ*_ON_ states evaluation.

We propose, instead, that the role of the oxygen vacancy migration and its coupling with the ferroelectric polarization of HZO can explain the thermal behavior shown in Figure 4. The mechanism is illustrated in Figure 4 bottom panels and summarized next, following the sketches from (1) to (4) clockwise. (1) Applying a large enough negative electric field, larger than the coercive field, from the top Au electrode switches the out‐of‐plane component of the polarization of the HZO layer towards it; at the same time, the positively charged oxygen vacancies migrate from the YSZ reservoir through the YSZ/HZO interface increasing the density of point defects and thus severely reducing the *κ* of HZO. (2) Note that the non‐reactive Au top electrode ensures that the oxygen vacancies stay in the HZO layer, and the ferroelectric polarization of the oxide retains the vacancies against diffusion when the electric field is removed. (3) In fact, the concentration of oxygen vacancies, and hence *κ*, remains stable upon further increases of the electric field towards positive values, until applying sufficient positive voltage (above the coercive field) which switches the polarization towards the YSZ substrate. At this point, oxygen vacancies rapidly migrate towards the YSZ, greatly increasing the *κ* of HZO as point defects density is reduced. (4) The out‐of‐plane component of the polarization of HZO pointing towards the YSZ inhibits oxygen vacancies from diffusing back to HZO, keeping the high values of *κ* of HZO even when the electric field is removed. Negative electric fields, larger than the coercive field, are needed to switch back the polarization towards the top Au metal, which allow oxygen vacancies to migrate to HZO, recovering the low *κ* value of HZO found in (1) and closing the *κ*(*E*) hysteresis loop. Therefore, the coupling between the polarization and the oxygen vacancy migration allows the precise and hysteretic tuning of *κ* in hafnia‐based epitaxial films. Although this coupling between polarization and oxygen ion migration was also proved in epitaxial ferroelectric BaTiO_3_ films embedded between a topotactic oxide (SrCoO_x_) and an ionic liquid [61], and, to a certain extent, in BaTiO_3_ epitaxial films grown on Nb:SrTiO_3_ [62], it has never been used as a mechanism to electrically manipulate *κ*. Ionic control of the metal‐oxide TBC in Pt/SrTiO_3_/Nb:SrTiO_3_ devices was recently reported, although in this case the absence of polarization results in spontaneous migration of oxygen vacancies against the chemical potential gradient and a gradual relaxation of the thermal states [48].

Previous work by Nukala et al., [53]. already proved that epitaxial HZO films behave as a sink of oxygen vacancies when negative voltage is applied through oxygen‐non‐reactive top Au electrodes. Therefore, we expect our epitaxial HZO films with the same top Au electrodes to behave likewise, giving rise to the situation (1) in Figure 4 bottom panels. However, the redistribution of oxygen vacancies induced by the ferroelectric polarization of HZO may involve not only the HZO layer itself but also the immediate interfacial region of the YSZ substrate. Note that during high‐temperature electrical poling, oxygen vacancies in the YSZ are mobile and can plausibly redistribute near the HZO/YSZ interface to partially screen the polarization bound charges of the HZO film. In this context, the formation of vacancy accumulation or depletion regions in YSZ—situation (2) and (4) in Figure 4 bottom panels—, electrostatically coupled to the polarization state of HZO, could additionally contribute to the observed changes in TBC. Still, electric‐field dependent FDTR experiments on Au/YSZ structures show no hysteretic electric‐field dependence of thermal transport (see Figure S4), demonstrating that oxygen vacancy redistribution in YSZ alone is insufficient. Moreover, in the non‐ferroelectric monoclinic HZO samples—Au/HZO_m_/YSZ—the absolute *κ* values of the HZO_m_ layers are similar to those of the ferroelectric HZO layer, yet the *κ*(*E*) dependence is fundamentally different. The ferroelectric HZO layer exhibits a clear, hysteretic *κ*(*E*) response with substantial modulation (Figure 4), whereas the non‐ferroelectric HZO_m_ layer displays a nearly field‐independent thermal conductivity (Figure S5). Thus, the mere presence of a hafnia‐based layer is not sufficient to induce an electric‐field‐dependent modulation of the TBC or thermal conductivity. Instead, the effect emerges only when the HZO layer is ferroelectric. This comparison between ferroelectric and non‐ferroelectric HZO therefore establishes ferroelectric polarization as a necessary ingredient to enable the observed hysteretic thermal response and evidences that the ferroelectric HZO layer governs the electrically controlled thermal transport in the Au/HZO/YSZ heterostructure.

**FIGURE 5:**
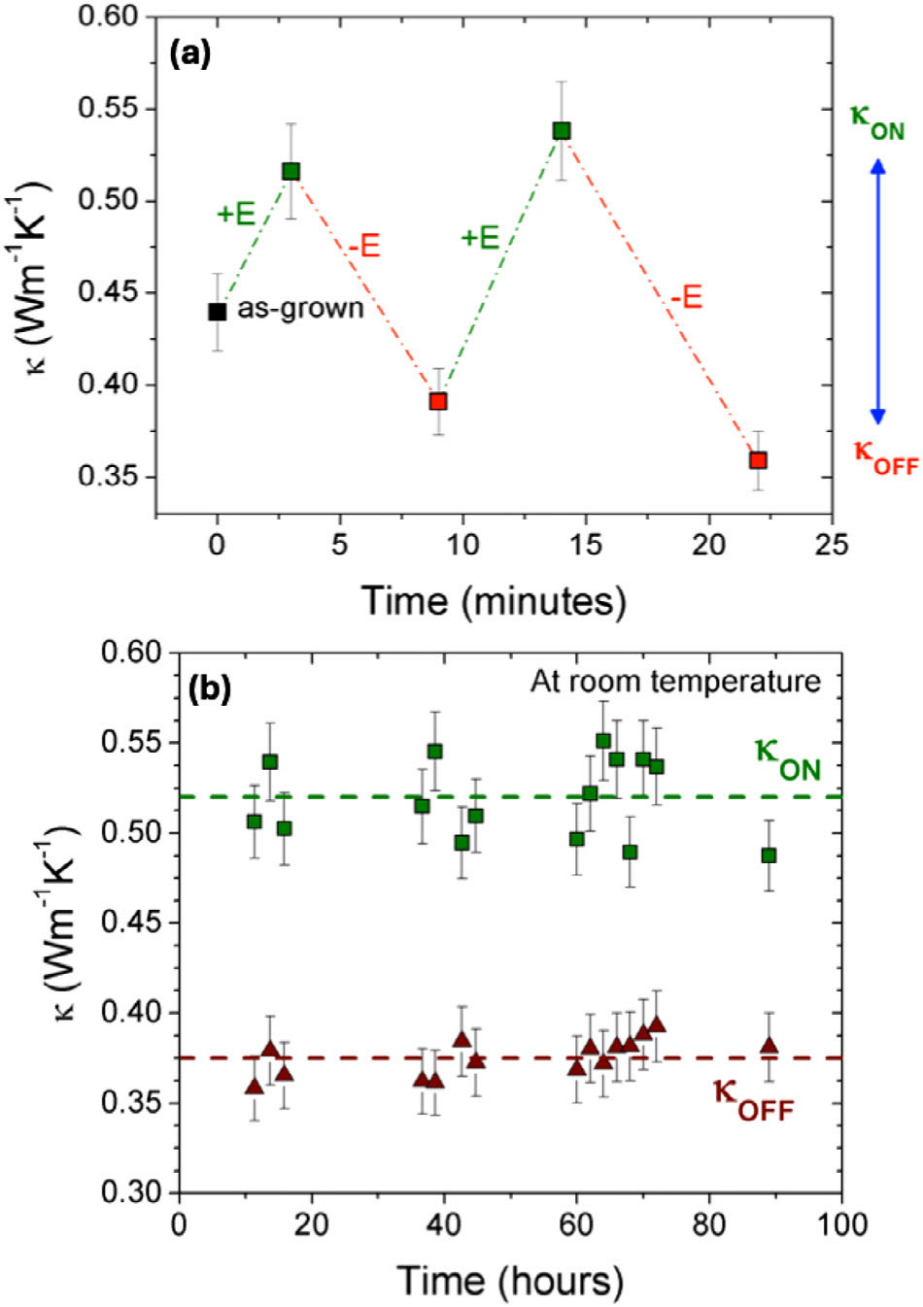
(a) *κ*_ON_/*κ*_OFF_ switching by applying electric fields with values of ±5.7·10^6^ V cm^−1^ (±E in the figure) at 200°C. (b) Stability of the *κ*_ON_ and *κ*_OFF_ values at room temperature with no electric field applied.

The *κ*_ON_ and *κ*_OFF_ states are reproducible, allowing us to recover the same thermal states after successive switching at 200°C with the same ± electric fields (Figure 5a), obtained by applying ± 4 V and providing a *κ*_ON_/*κ*_OFF_ ratio ≈ 1.6. To evaluate the robustness of the *κ*_ON_ and *κ*_OFF_ states, the HZO film was polarized under positive/negative electric fields at 200°C. Then, while still applying the field, the film was cooled down to room temperature. Once that point was reached, the electric field was removed and the thermal conductivity of the ON/OFF states as a function of time was tracked. Note that cooling the sample under applied electric field stabilizes the interfacial charge distribution (vacancy depletion/accumulation regions in the YSZ near the HZO/YSZ interface) that remains coupled to the polarization state even after the external field is removed, preventing the depolarization of the film. As shown in Figure 5b, the time dependence of the *κ*_ON_ and *κ*_OFF_ values is stable for several days under laboratory conditions. Notice that the fact that these values are almost the same of those at 200°C reported in Figure 4 is consistent with the preponderance of temperature‐independent scattering mechanisms (boundaries and disorder) in contrast to temperature‐dependent anharmonic scattering, as reported in nanometric‐thick films and other nanostructured materials [46, 63, 64]. The stability of the ON/OFF states at room temperature was found to be reproducible using several Au pads in the same sample, as well as in different samples. This confirms that spontaneous diffusion of oxygen vacancies is strongly suppressed at room temperature. The reported diffusion coefficients of oxygen (or oxygen vacancies) in hafnia‐based thin films at room temperature span several orders of magnitude. For instance, values as low as ≈10^−26^ cm^2^ s^−1^ have been reported in 12.7 nm thick polycrystalline HZO films, with a thermal activation energy of ≈1 eV [65]. In contrast, much larger diffusion coefficients, ≈10^−14^ cm^2^ s^−1^, were reported for 4 nm thick HfO_2_ films, with a lower activation energy of ≈0.5 eV [66]. In epitaxial films, a very fast oxygen ion migration has been reported at room temperature for ≈6 nm‐thick ferroelectric HZO films—comparable in thickness to the films studied here—, even though a quantitative diffusion coefficient was not explicitly extracted [53]. For intermediate diffusion coefficients of oxygen ions (≈10^−17^ cm^2^ s^−1^) as the ones reported for epitaxial SrTiO_3_ films, oxygen vacancies dragged to the surface by an applied electric field rapidly diffuse back into the bulk within a few hours at room temperature when the applied voltage is removed [67]. In contrast, the thermal conductivity states shown in Figure 5b remain stable for tens of hours (at least) at room temperature. If the stationary states were governed solely by kinetic limitations of oxygen vacancy diffusion, a comparable relaxation timescale would be expected, as observed in other epitaxial oxide systems. The much longer stability observed here therefore suggests that kinetics alone cannot account for the stationary behavior of oxygen vacancies in our HZO films. Instead, we infer that the stabilization of these states requires the coupling between oxygen vacancies migration and the ferroelectric polarization, which provides an additional electrostatic mechanism to sustain non‐equilibrium vacancy distributions over extended times.

**FIGURE 6:**
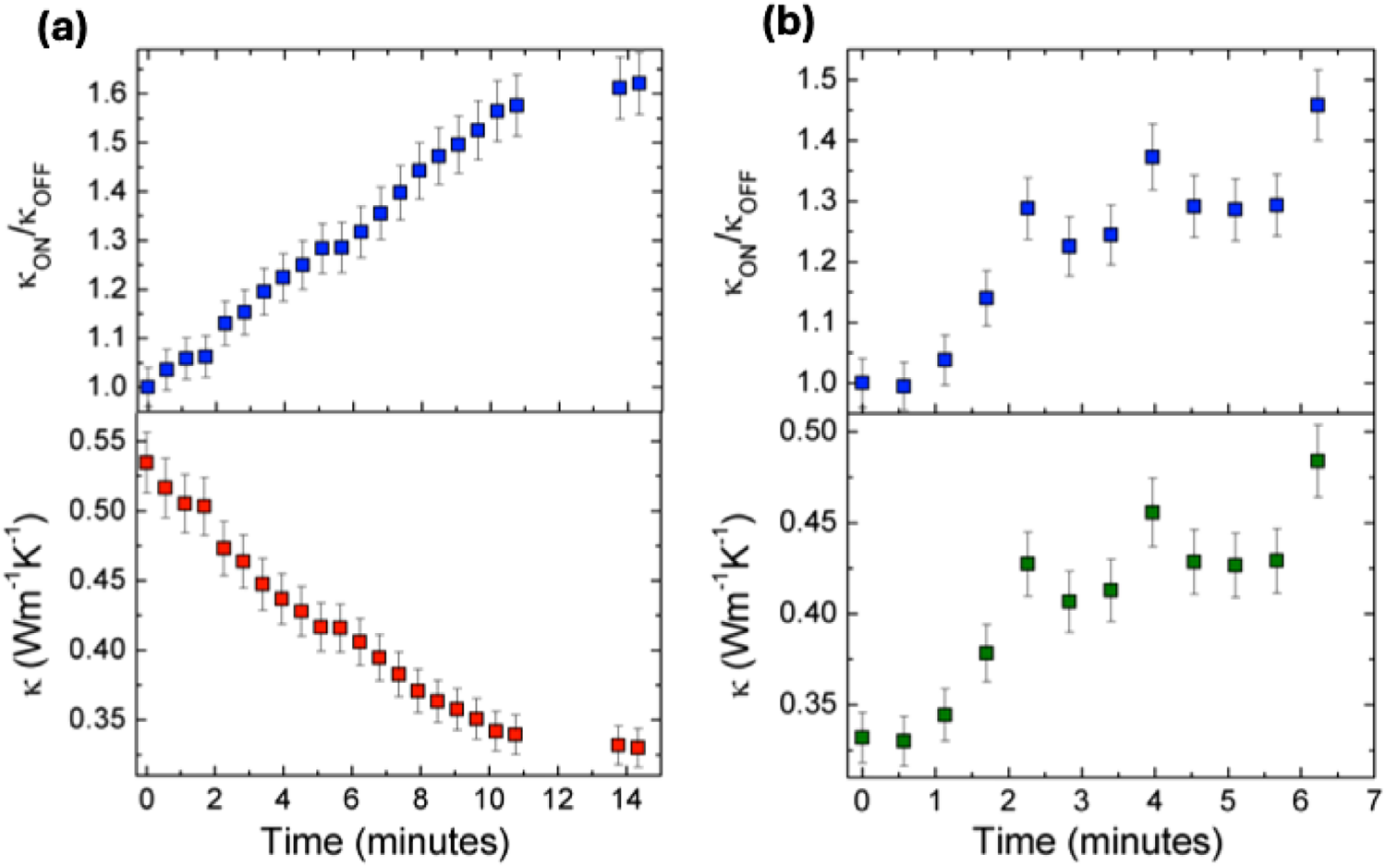
κ_ON_/κ_OFF_ ratio (top panels) and thermal conductivity (lower panels) as a function of time when, at 200°C, the applied voltage is immediately switched (a) from +3 to ‐3 V, and (b) from ‐3 to +3 V.

Regarding the speed of the process, Figure 6 shows the evolution of the *κ* of HZO film with time after switching from ‐3 to +3 V and vice versa. As previously noted, it takes several minutes to reach the maximum *κ*_ON_/*κ*_OFF_ ratio, which signals the time required to reach the equilibrium population of oxygen vacancies between the film and the YSZ reservoir. A much larger oxide‐ion mobility has been reported in HZO at room temperature when grown on Nb:SrTiO_3_ substrates [53], and the process can be expected to be even faster at 200°C; we thus conclude that the *κ*_ON_/*κ*_OFF_ switching time in our samples could be limited by the ion mobility at the YSZ/HZO interface or in the YSZ substrate itself. In a previous work a faster oxygen ion mobility in YSZ was achieved by increasing not only the temperature (at 280°C), but also the applied voltage (8 V) [20]. However, in the Au/HZO/YSZ samples, either the increase in temperature or the applied electric field resulted in a rapid deterioration of the Au/HZO interface, detaching the Au layer from the HZO film, which impeded the FDTR and the electrical measurements.

The oxygen‐vacancy‐driven reduction of *κ* highlighted by our experiments and discussed above is corroborated by theoretical calculations based on the Boltzmann transport equation (BTE) [68], where both phonon‐phonon anharmonic scattering up to the third order and phonon‐vacancy scattering are described within an entirely ab initio framework, and specifically the effect of vacancies is accounted for using an atomistic Green's function formalism with proven predictive accuracy (see the Supporting Information for full details) [69, 70, 71]. Our computed *κ* for bulk Hf_0.5_Zr_0.5_O_2_ at 300 K agrees well with the results of Zhang and co‐workers [59], i.e., 8–9 W m^−1^ K^−1^. According to their estimates, this value is then reduced i) by mass disorder, down to 3.9 W m^−1^ K^−1^, and ii) by boundary scattering, reaching 1.8 W m^−1^ K^−1^, if a film thickness of 20 nm is considered. While, for reasons of computational cost, in our calculations we did not explicitly account for sources of disorder other than vacancies, we estimated the reduction of *κ* for a 7 nm thin film, like the one considered in our experiments, to be of around 80% (see the Supporting Information). When oxygen vacancies are present, the thermal conductivity is further reduced by defect scattering. At 200 K, we obtain a range of *κ*_ON_/*κ*_OFF_ ratios between 1.23 and 5.70 when the oxygen vacancy densities are varied from 7·10^19^ to 2.25·10^21^ cm^−3^, typical of HfO_2_‐based samples of this type. In particular, the ≈1.6 experimental ratio is predicted to be achieved with a vacancy density of ≈2.2·10^20^ cm^−3^ (see Figure S7). This corresponds to ≈0.03 oxygen vacancies per unit cell—or 3 vacancies in 100 unit cells. Therefore, relatively low density of point defects in HZO is capable to significantly scatter phonons.

It is worth noting that in measuring the electric‐field dependence of the TBC and extracting *κ*(*E*), we consider that the heat capacity, *C*_p_, remains unaltered. In the FDTR measurements, at low modulation frequencies, the system operates in the quasi‐steady‐state diffusion regime, where the thermal penetration depth is large and the temperature field equilibrates within each modulation cycle [72]. If *C*_p_ were varying with electric field, this would primarily affect the low‐frequency FDTR response, where heat storage dominates [72]. The absence of any measurable divergence or hysteresis at low frequencies in our data strongly argues against a significant field‐induced change in *C*_p_. Moreover, the different values of *C*_p_ of the hafnia‐based layer (namely, in the high‐ and in the low‐conductivity state) are not expected to change significantly. First, no phase transition is driven in our HZO films during the electric field cycling (Figure 2) which would be susceptible to changes in *C*_p_ [73]. Second, the impact of point defects, such as vacancies (i.e., the scattering centers responsible for the two conductivity states observed in out experiments), have usually negligible effect on *C*_p_, as long as their concentration is small, which is certainly the case in our hafnia‐based samples as discussed in the theoretical calculations. In particular, Li et al. found that point defects in β‐SiC have essentially no effect on the heat capacity [74]. This observation matched well with the general understanding that point defects in low concentration mostly scatter short wavelength, high‐frequency phonons [75], so that the low‐frequency portion of the density of states is essentially unaltered. Similarly, Senor et al. reported the insensitivity of *C*_P_ values to point defects in low‐impurity samples and showed that attempts to calculate specific heats using the Debye model for various SiC‐based composites containing differing amounts of impurities gave virtually indistinguishable results [76]. Third, in ferroelectrics, pronounced *C*_p_ anomalies are observed at temperature‐driven ferroelectric–paraelectric phase transitions, but not during polarization switching within the ferroelectric phase [77]. At temperatures close to the ferroelectric‐paraelectric phase transition temperature, where the pronounced peak in *C*_p_ is found and the spontaneous polarization approaches to zero, *C*_p_ may strongly be electric‐field dependent, generally reducing the magnitude of the *C*_p_ peak and shifting it towards higher temperatures [78, 79, 80]. However, this reduction of *C*_p_ is not expected to depend on the polarity of the electric field. Consequently, *C*_p_(*E*) would exhibit a symmetric response with respect to positive and negative fields, rather than the pronounced hysteretic behavior observed in our measurements (Figure 4). But, more importantly, at temperatures below that of the phase transition, in the ferroelectric regime, the electric‐field dependence of *C*_p_ is quite negligible [78, 79, 80]. This behavior is well established experimentally and in the context of electrocaloric studies, where significant electric‐field‐induced thermal changes occur only around the ferroelectric‐paraelectric phase transition [81, 82, 83].

## 3 Conclusions

In summary, we demonstrate the potential of ferroelectric Hf_1‐x_Zr_x_O_2_ as a thermally tunable material for solid‐state thermal memory applications. The findings reveal that the electric‐field dependence of thermal conductivity in HZO films exhibits a hysteresis loop, resembling their polarization‐electric field behavior, which enables the definition of non‐volatile *κ*_ON_ and *κ*_OFF_ states in the absence of applied voltage with a *κ*_ON_/*κ*_OFF_ ratio of 1.6. The coupling of oxygen vacancy migration with ferroelectric polarization was identified as the primary mechanism influencing thermal conductivity, offering an alternative to phonon scattering via electric‐field‐reconfigurable domain walls. On the other hand, the *κ*_ON_ and *κ*_OFF_ thermal states proved to be highly stable, retaining their values over days, which suggests minimal oxygen vacancy rediffusion at room temperature. However, the *κ*_ON_/*κ*_OFF_ switching time was slower than anticipated, primarily due to limitations in ion mobility within the YSZ substrate, despite the rapid mobility previously reported in the HZO layer itself. In addition, fatigue studies deserve future work aimed at assessing long endurance in the *κ*_ON_/*κ*_OFF_ switching cycles. Overall, this work on HZO films presents a new viable approach for actively modulating the thermal conductivity, which opens a new pathway in the engineering of electrically controlled thermal memory devices.

## 4 Experimental Section

### 4.1 Epitaxial Growth and Structure Characterization of Hf_0.5_Zr_0.5_O_2_ Films

HZO thin films (7 nm thick) were grown on 111‐oriented and on 001‐oriented YSZ single‐crystal substrates (Crystal GmbH, with nominally 9.5 mol% Y_2_O_3_ and lattice parameter *a* = 5.15 Å) by pulsed laser deposition (PLD). The growth was carried out at a substrate temperature of 850°C, a dynamic O_2_ pressure of 100 mTorr, and a fluence of 1 J cm^−2^ using a KrF laser operated at a repetition rate of 10 Hz. After deposition, the films were cooled down to at 10°C min^−1^ under the same oxygen pressure as during growth. The crystal structure and thickness of the films were assessed by high‐resolution X‐ray diffraction (XRD) and X‐ray reflectivity (XRR), respectively, using a Bruker D8 Advance diffractometer equipped with parallel‐beam optics and monochromated Cu‐Kα_1_ radiation (wavelength *λ* = 1.54056 Å).

Atomic‐scale characterization of the Au/HZO/YSZ heterostructure in the ON and OFF state was performed by scanning transmission electron microscopy (STEM). High‐angle annular dark field (HAADF) imaging was carried out in a Thermo Fisher Titan Low Base 60–300 electron microscope equipped with a high‐brightness Schottky field emission gun and a CETCOR probe‐corrector (CEOS Gmbh) and operated at 300 kV. A cross‐sectional lamella of the specimen was cut along (110) plane of the 111‐oriented YSZ substrate by Focused Ion Beam milling in a Thermo Fisher Helios 650 Nanolab. A HAADF‐STEM image simulation of the orthorhombic HZO structure was carried out with the Dr. Probe software package [84].

### 4.2 Thermal Conductivity Measurements

The electrical and temperature dependence of cross‐plane thermal conductivity of the HZO films was measured by commercial Frequency Domain Thermoreflectance equipment (Fourier Scientific, LLC) in a temperature‐dependent electrical probe station (Instec). 60 nm thick Au metal layer—the thermoreflectance transducer—is deposited on top of the HZO/YSZ samples by sputtering (GATAN 682 Precision Etching and Coating System). A sinusoidally modulated pump laser (*𝜆* = 488 nm, modulating *f* = 2 kHz– 50 MHz, spot sizes 1/*e* ^2^ radius ≈3.7 or 10.5 µm) was focused on the Au layer to produce an oscillatory modulation of the surface temperature. This results in a periodic variation of the Au thermoreflectance, which was probed by a laser beam (*𝜆* = 532 nm). The thermal properties of the sample were obtained by fitting the phase data to an analytical solution of the heat diffusion equation in a multilayer model (see Supporting Information for further details).

### 4.3 Electrical Measurements

Current–electric field measurements were carried out at high temperature (185°C) and low frequency (0.01 Hz), conditions under which the YSZ substrate behaves as an effective conductive element due to its thermally activated oxide‐ion conductivity. Voltage sine waves of increasing amplitude were applied using an HP3325B function generator, and the resulting current response was measured with a home‐built charge‐to‐voltage converter. The total current was decomposed into conductive, linear dielectric, and ferroelectric switching contributions, and the ferroelectric switching current was isolated following the fitting procedure described in ref. [85].

### 4.4 Computational Details

The harmonic and anharmonic interatomic force constants (IFCs) for Hf_0.5_Zr_0.5_O_2_ were computed using a finite‐displacement scheme applied to a 3 × 3 × 3 supercell of the orthorhombic primitive cell. The pristine and defect‐laden supercells contain 324 and 323 atoms, respectively. To model the defective structure, a single oxygen atom is removed from the supercell. An additional relaxation of the atomic positions is then performed with the cell volume held fixed, prior to the harmonic IFC calculation.

All first‐principles calculations, including structural relaxations and force evaluations for the IFCs, were conducted using the projector‐augmented‐wave (PAW) method [86] as implemented in the VASP density functional theory package [87], employing the PBE exchange‐correlation functional [88]. A plane‐wave energy cutoff of 600 eV and a 3 × 3 × 3 Monkhorst‐Pack **k**‐point mesh were used for structural relaxations. For the force calculations on the displaced structures, a single **k**‐point at the center of the supercell Brillouin zone was employed. The relaxed lattice parameters of the pristine Hf_0.5_Zr_0.5_O_2_ primitive cell are *a* = 5.029 Å, *b* = 5.049 Å, and *c* = 5.233 Å.

Second‐ and third‐order IFCs were extracted using the Phonopy package [89] and the thirdorder.py code [90], respectively. To account for the non‐analytical correction to the dynamical matrix—required for an accurate description of LO‐TO phonon splitting—Born effective charges and the dielectric tensor were computed using VASP and included in the analysis.

Defect scattering was evaluated via Green's function calculations performed on a 13 × 13 × 13 grid, using the tetrahedron method to integrate over the Brillouin zone [91] and a real‐space cutoff radius of 8.5 Å. All scattering rates were calculated on a 12 × 12 × 12 **q**‐point mesh.

The computation of Green's functions, scattering rates, and final thermal conductivity tensor was carried out using the almaBTE code, developed by one of the authors [68].

## Author Contributions

E.L. initiated and supervised the project together with F.R., J.A.P., and R.R. D.B. and H.R.‐B. grew the epitaxial HZO films by PLD. H.R.‐B. and J.A.P. performed the XRD and XRR measurements. D.B., R.R., V.L., and N.V.‐D. carried out the FDTR measurements. M.R, J.C., and R.R. performed the theoretical calculations. C.M. carried out the STEM characterization. R.J. and M.A. performed the electrical measurements. E.L. wrote the paper with contributions from the rest of the authors.

## Conflicts of Interest

The authors declare no conflicts of interest.

## Supporting information




**Supporting File**: adma72921‐sup‐0001‐SuppMat.docx.

## Data Availability

The data that support the findings of this study are available from the corresponding author upon reasonable request.
