# Supplementary material for: The Coupling of Ferroelectric Polarization and Oxygen Vacancy Migration Enables Electrically Controlled Thermal Memories
Source: Adv Mater. 2026 Mar 30;38(24):e19670. doi: 10.1002/adma.202519670 (PMC13113234; doi:10.1002/adma.202519670)
Supplement: Supplementary file 1 — Supporting File: adma72921‐sup‐0001‐SuppMat.docx. [file ADMA-38-e19670-s001.docx]

**Supplementary information:**

**The coupling of ferroelectric polarization and oxygen vacancy migration enables electrically controlled thermal memories.**

Dídac Barneo^1,2^, Miquel Royo^3^, Rafael Ramos^4,5^, Jesús Carrete^6,7^, Hugo Romero-Bernad^6^, Ricardo Jiménez^8^, Víctor Leborán^4^, César Magén^6,7^, Noa Varela-Domínguez^4,5^, Miguel Algueró^8^, Riccardo Rurali^3^, José A. Pardo^6,9,10^, Francisco Rivadulla^4,5^, Eric Langenberg^1,2,^*

^1^Departament de Física de la Matèria Condensada, Universitat de Barcelona, 08028 Barcelona, Spain.

^2^Institut de Nanociència i Nanotecnologia (IN^2^UB), Universitat de Barcelona, 08028 Barcelona, Spain.

^3^Institut de Ciència de Materials de Barcelona, ICMAB-CSIC, Campus UAB, 08193 Bellaterra, Spain,

^4^Centro Singular en Química Biolóxica e Materiais Moleculares (CiQUS), Universidade de Santiago de Compostela, 15782 Santiago de Compostela, Spain.

^5^Departamento de Química-Física, Universidade de Santiago de Compostela, 15782-Santiago de Compostela, Spain.

^6^Instituto de Nanociencia y Materiales de Aragón (INMA), CSIC-Universidad de Zaragoza, 50009 Zaragoza, Spain.

^7^Departamento de Física de la Materia Condensada, Universidad de Zaragoza, 50018 Zaragoza, Spain.

^8^Instituto de Ciencia de Materiales de Madrid (CSIC), 28049 Madrid, Spain.

^9^Departamento de Ciencia y Tecnología de Materiales y Fluidos, Universidad de Zaragoza, 50018 Zaragoza, Spain.

^10^Laboratorio de Microscopías Avanzadas, Universidad de Zaragoza, Campus 
Río Ebro, 50018 Zaragoza, Spain

**Structural characterization.**

The crystallographic orientation of the Y_2_O_3_:ZrO_2_ (YSZ) substrates is determinant when it comes to stabilizing either the non-polar monoclinic phase (favored by the 001-orientation) or the polar orthorhombic phase (favored by the 111-orientation) in the Hf_0.5_Zr_0.5_O_2_ (HZO) epitaxial films.^[1]^ Hereinafter HZO_m_ and HZO_FE_ stand for the non-polar and polar phase, respectively. Two HZO films–7 nm thick–were simultaneously grown onto 001- and 111-oriented YSZ substrates by pulsed laser deposition (see Experimental Section). As shown in the θ/2θ X-ray diffraction patterns (Fig. S1), the HZO peak appears at 2θ ≈ 30.7° for HZO_FE_/YSZ(111), consistent with the polar phase, while it shifts to 2θ ≈ 34.1° for HZO_m_/YSZ(001), characteristic of the monoclinic phase.


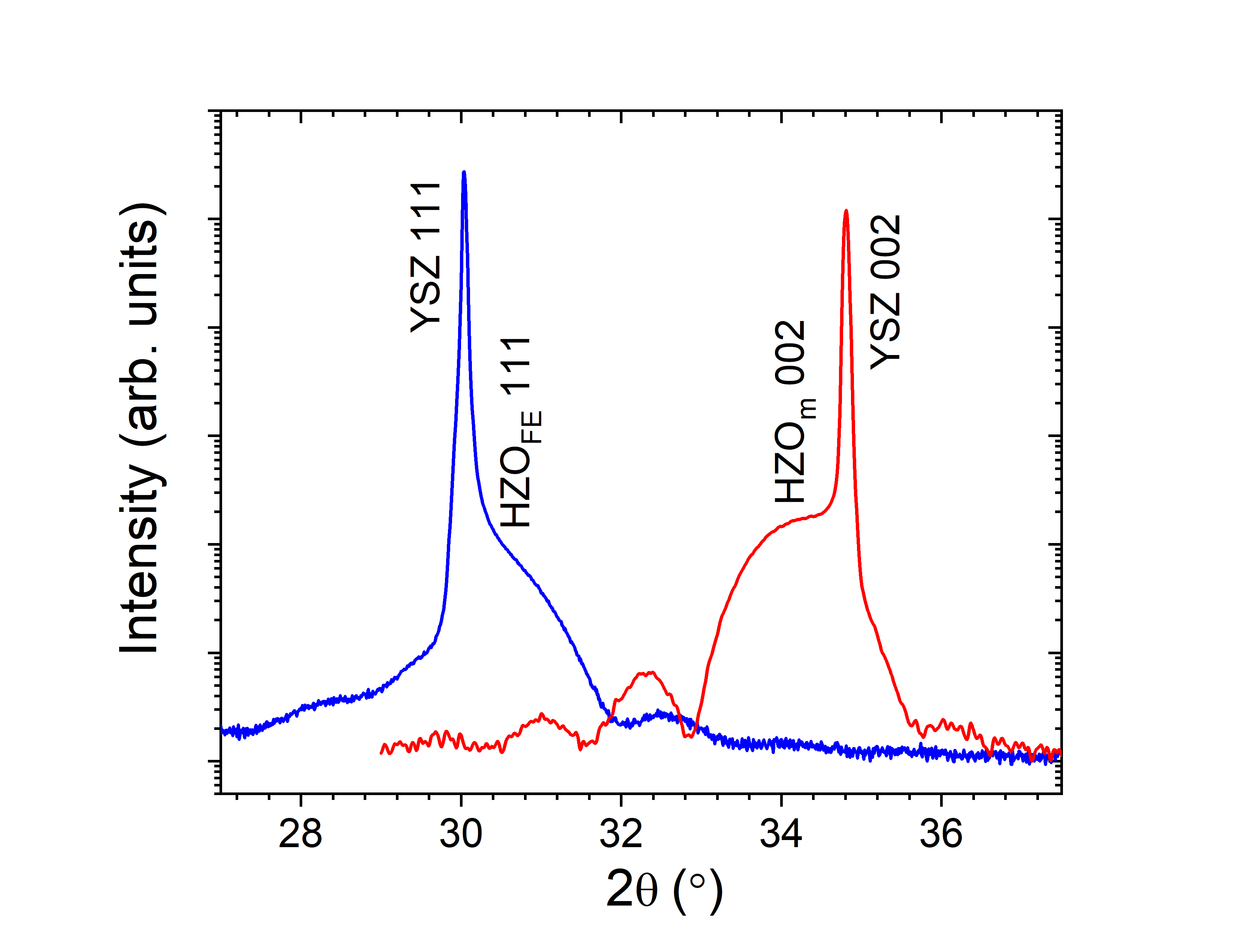


***Fig. S1****. θ/2θ X-ray diffraction pattern of Hf_0.5_Zr_0.5_O_2_ films deposited onto 111-oriented YSZ (blue line) and 001-oriented YSZ (red line) substrates.*

**Thermal conductivity measurements.**

Thermal characterization was done by Frequency-Domain Thermoreflectance (FDTR).^[2,3]^ FDTR is a non-contact optical pump–probe technique. It fundamentally works with two different laser beams: the first beam of light (the pump) acts as the heat source while the second one (the probe) detects the temperature change caused by the pump and the thermal dissipation (∆𝑇) through a change in surface reflectivity (∆𝑅): ∆𝑇 = (𝑑𝑅/𝑑𝑇)^−1^∆𝑅 = (𝛽)^−1^∆𝑅. In order to properly quantify this reflectance (and its changes), a transducer is normally deposited on top of the studied materials, normally Au, and in our system it was used as top electrode as well. Measurements are performed in a wide frequency range at constant irradiation, making a sweep in frequency, differently from Time-Domain Thermoreflectance where the variable magnitude is the time of the pulse from the laser (period). The variable heat source produces temperature gradients and transients which enables the measurement of the thermal boundary conductance (TBC) between Au and YSZ substrate–or the equivalent HZO film as explained later–with good precision.

In our setup (Fig. S2a), a sinusoidal modulated pump laser (λ=405 nm, modulating frequency 2 kHz–50 MHz, Gaussian spot size 1/e^2^ radius ≈ 10.5 μm) is focused on the surface of the film, coated with a 60-nm-thick layer of Au. This sinusoidal irradiation produces an oscillatory modulation of the temperature in the surface, and therefore a periodic change of the Au thermoreflectance. The laser beam used to probe these phenomena (λ=532 nm) is split to measure both the signal before reaching the sample (reference) and after, getting a variation in the phase between both parts of the beam. Also, this process is used to improve the signal-to-noise ratio, especially at low frequencies, and calibrate well the phase-shift offsets produced by electronics and differences between optic paths.

Then, this phase shift measured in the stablished frequency range is fitted to an analytical solution of the heat diffusion equation for layered structures to determine the total thermal resistance (*R*_Tot_). ^[3]^ More specifically, a multilayer model consisting on an Au film on top of an YSZ film is used, and the κ of the HZO film is obtained by considering it a thermal resistance, included in the TBC between Au and YSZ. This is done in this way because the HZO films are very thin compared to the other two materials, so the variations they produce in measurements are sufficiently small to be simplified for less complex (and more realistic) mathematical analysis. The model used is sketched in Fig. S2b.


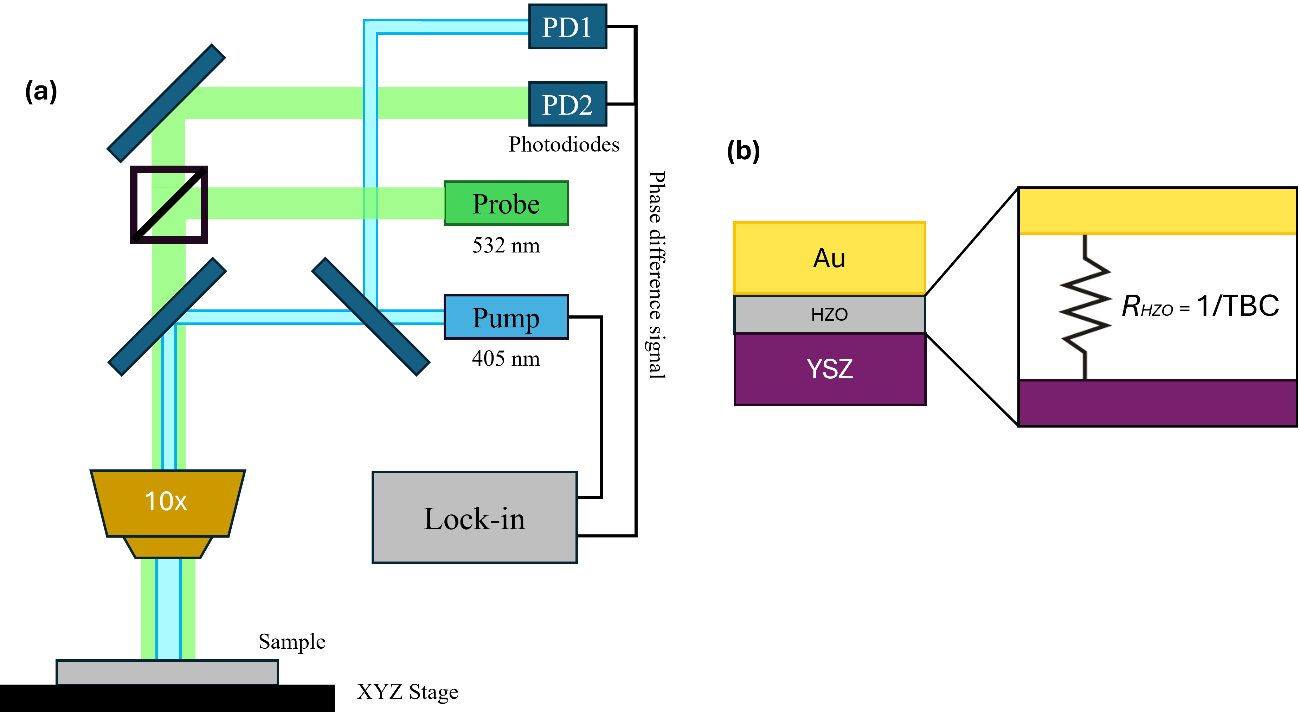


***Fig. S2.*** *Simplified schemes of* ***(a)*** *the FDTR setup and* ***(b)*** *the multilayer model used to fit the FDTR data, considering the HZO film as a thermal resistance.*

The specific heat capacity values, Cp, used for the fitting were extracted from the literature for YSZ and Au,^[4,5]^ as well as the thermal conductivity of YSZ.^[6]^ κ of Au was obtained by measuring its electrical conductivity, and applying the Wiedemann-Frantz law to obtain its thermal counterpart. YSZ thickness is taken from the nominal value (0.5 mm) provided by the supplier (Crystal GmbH), and the thickness of the Au transducer layer was quantified by X-ray reflectivity. Each parameter has different sensitivities upon frequency range.

***Table S1.*** *Parameters used for fitting the FDTR data*

|  | Cp  (MJ·K^-1^·m^-3^) | κ⊥  (W·m^-1^·K^-1^) | Thickness  (nm) | TBC  (W·m^-2^·K^-1^) |
| --- | --- | --- | --- | --- |
| Au transducer | 2.2 | 90 | 60 |  |
|  |  |  |  | Fitting Parameter |
| YSZ substrate | 2.8 | 2.5 | 50000 |  |
|  |  |  |  |  |

***Fig. S3.*** *Frequency dependent phase (φ) data (dots) of the FDTR measurements in* Au/HZO_m_/YSZ (top panel) and Au/HZO_FE_/YSZ (bottom panel) *samples at different electric fields at 200ºC. The solid lines correspond to the fitting to the analytical solution of the heat diffusion equation for layered structures. The frequency range where the effects of TBC are maximized is pictured in the figure on the right side of each panel.*


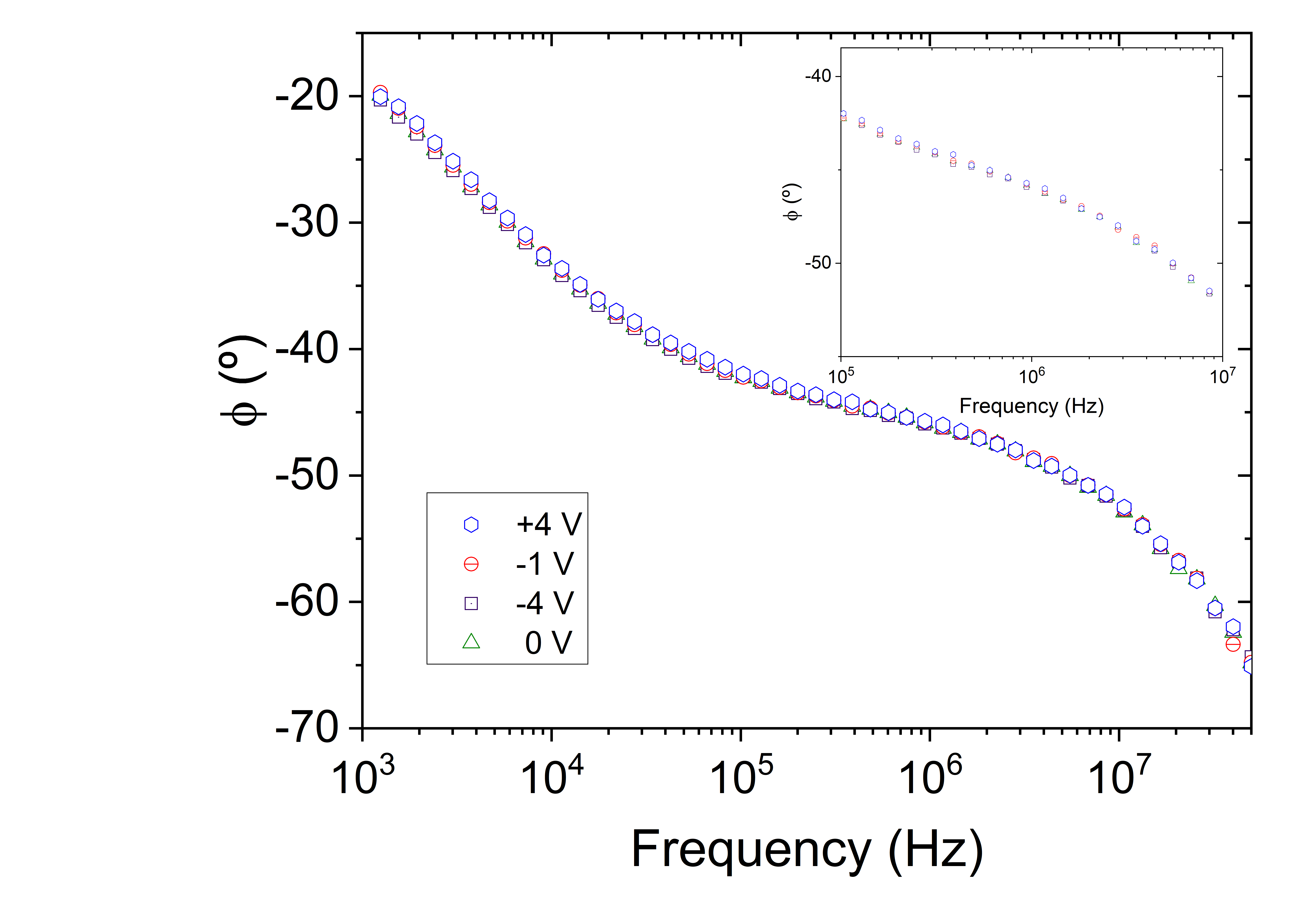


***Fig. S4.*** *Frequency dependent phase (φ) data of the FDTR measurements in Au/YSZ samples at different electric fields at 200ºC. The frequency range where the effects of TBC are maximized is shown in the inset.*

***Fig. S5****. Electric-field dependence of the thermal conductivity of Hf_0.5_Zr_0.5_O_2_ films in the ferroelectric phase (blue squares) and monoclinic phase (red circles) at 200ºC obtained from the fitting to the analytical solution of the heat diffusion equation (solid lines in Fig. S3).*

**Electrical Measurements.**


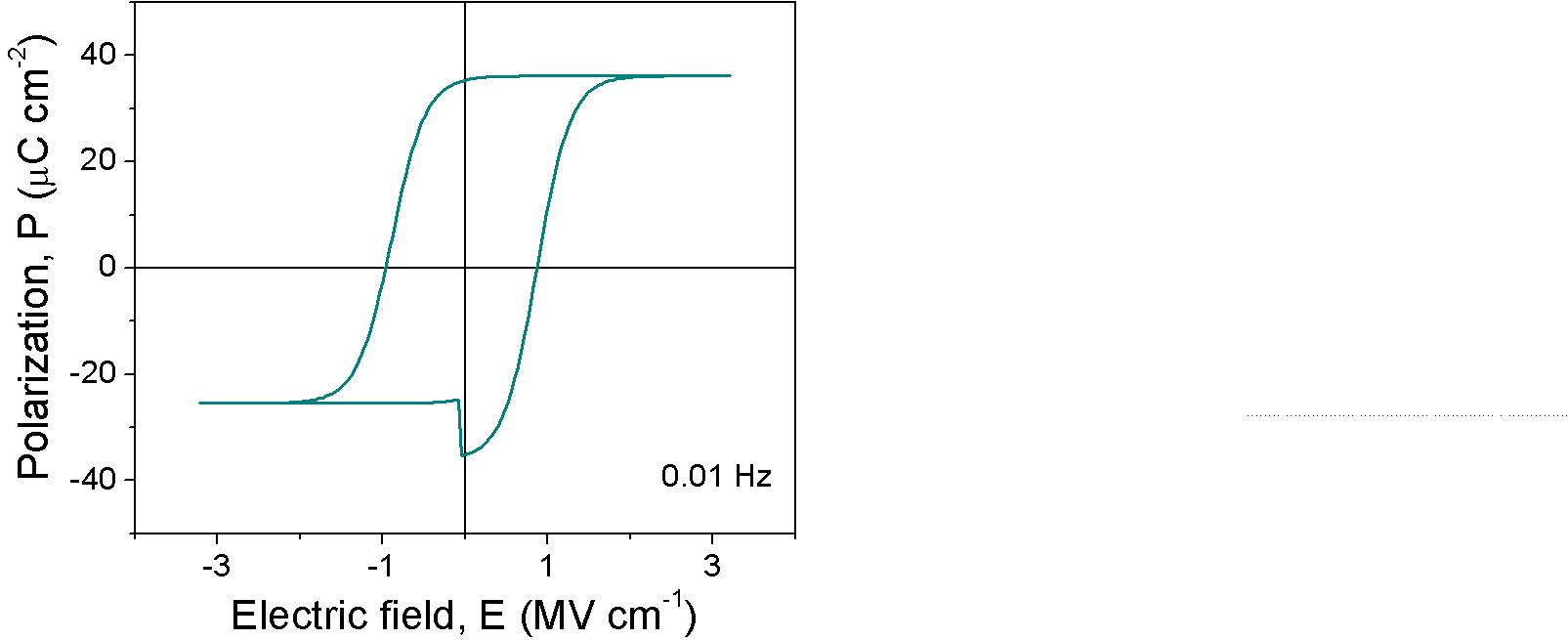


***Fig. S6.*** *Polarization-Electric-field, P(E), hysteresis loop is reconstructed from the ferroelectric switching current (Fig. 4 in main text)*

**Lattice thermal conductivity calculations.**

The isotropic phonon-contributed thermal conductivity was calculated, in the relaxation-time approximation, as

$$\kappa_{\mathcal{l}}=\frac{1}{3k_{B}T^{2}V}\sum_{\lambda} f_{0\lambda}\left( f_{0\lambda}+1 \right)\left( \hbar\omega_{\lambda} \right)^{2}v_{\lambda}^{2}\tau_{\lambda}$$

Here, $k_{B}$ is the Boltzmann constant, $T$ is the temperature, and $V$ is the unit cell volume; $\lambda$ runs over all phonon modes (indexed by their wave vector and phonon branch) and $\omega_{\lambda}$, $v_{\lambda}$, $f_{0\lambda}$ and $\tau_{\lambda}$ are the angular frequency, group velocity, equilibrium (Bose-Einstein) occupancy and lifetime, respectively.

The harmonic quantities $\omega_{\lambda}$, $v_{\lambda}$ (and therefore $f_{0\lambda}$) are extracted from the phonon spectrum, which in turn requires knowledge of the second-order derivatives of the potential energy of the system at its equilibrium configuration – the second-order force constants (IFCs). For the defect-free system, $\tau_{\lambda}$ contains contributions from isotopic disorder, evaluated based on the isotopic distribution of each element, and from anharmonic scattering, with the leading term of the latter being three-phonon scattering; computing the three-phonon matrix elements requires obtaining the third-order derivatives of the potential energy, or third-order IFCs, as well. All the details of the procedure are available in Ref. 7.

In the defect-laden system, elastic phonon-vacancy interactions constitute a third source of phonon scattering. Its contribution to the total scattering rate, $\tau_{\lambda}^{-1}$, is proportional to $-\text{Im}\left\{ \left\langle\lambda| \boldsymbol{t} | \lambda\right\rangle\right\}$ according to the optical theorem of perturbation theory, where the t matrix

$$\boldsymbol{t=}\left( \boldsymbol{1-Vg} \right)^{\boldsymbol{-1}}\boldsymbol{V}$$

combines the information about the perfect crystal hosting the vacancy (through its Green’s function $\boldsymbol{g}$) and about the perturbation itself (contained in $\boldsymbol{V}$, which measures the change in the harmonic IFCs causes by the vacancy). The procedure is detailed in Ref. 8.

***Fig. S7.*** *Calculated lattice thermal conductivity of* Hf_0.5_Zr_0.5_O_2_ *along the direction parallel to the dielectric polarization as a function of the oxygen vacancy density. Results are shown for two temperatures, 200 K and 300 K, represented by solid circles. Lines are included as guides to the eye.*

**References**

[1] E. Barriuso, R. Jiménez, E. Langenberg, P. Koutsogiannis, Á. Larrea, M. Varela, C. Magén, P. A. Algarabel, M. Algueró, J. A. Pardo, “Epitaxy-Driven Ferroelectric/Non-Ferroelectric Polymorph Selection in an All-Fluorite System,” *Advanced Electronic Materials* 10 (2024): 2300522, <https://doi.org/10.1002/aelm.202300522>

[2] A. J. Schmidt, R. Cheaito, M. Chiesa, “A frequency-domain thermoreflectance method for the characterization of thermal properties,” *Review of Scientific Instruments* 80 (2009): 094901, <https://doi.org/10.1063/1.3212673>

[3] D. J. Kirsch, J. Martin, R. Warzoha, M. McLean, D. Windover, I. Takeuchi, “An instrumentation guide to measuring thermal conductivity using frequency domain thermoreflectance (FDTR),” *Review of Scientific Instruments* 95 (2024): 103006, <https://doi.org/10.1063/5.0213738>

[4] A. V. Khvan, I. A. Uspenskaya, N. M. Aristova, Q. Chen, G. Trimarchi, N. M. Konstantinova, A. T. Dinsdale, “Description of the thermodynamic properties of pure gold in the solid and liquid states from 0 K,” *Calphad* 68 (2020): 101724. <https://doi.org/10.1016/j.calphad.2019.101724>

[5] E. I. Salamatov, A. V. Taranov, E. N. Khazanov, E. V. Charnaya, E. V. Shevchenko, “Transport characteristics of phonons and the specific heat of Y_2_O_3_:ZrO_2_ solid solution single crystals,” *Journal of Experimental and Theoretical Physics* 125 (2017): 768. <https://doi.org/10.1134/S1063776117100144>

[6] E. Langenberg, E. Ferreiro-Vila, V. Leborán, A. O. Fumega, V. Pardo, F. Rivadulla, “Analysis of the temperature dependence of the thermal conductivity of insulating single crystal oxides,” *APL Materials* 4 (2016): 104815. <https://doi.org/10.1063/1.4966220>

[7] W. Li, J. Carrete, N. A. Katcho, N. Mingo, “ShengBTE: A solver of the Boltzmann transport equation for phonons,” *Computer Physics Communications* 185 (2014), 185, 1747, <https://doi.org/10.1016/j.cpc.2014.02.015>

[8] B. Dongre, J. Carrete, A. Katre, N. Mingo, G. K. H. Madsen, “Resonant phonon scattering in semiconductors,” *Journal of Materials Chemistry C* 6 (2018): 4691, [https://doi.org/10.1039/C8TC0082](https://doi.org/10.1039/C8TC00820E)
